# Ethnic-Racial Identity and Attitude Change: Assessments of Outgroup and Diversity Attitudes among Adolescents in Sweden

**DOI:** 10.1007/s10964-024-02024-4

**Published:** 2024-06-04

**Authors:** David J. Sandberg, Ann Frisén, Linda P. Juang, C. Philip Hwang, Moin Syed

**Affiliations:** 1 Department of Psychology, University of Gothenburg, Gothenburg, Sweden; 2 Department of Inclusive Education, University of Potsdam, Potsdam, Germany; 3 Department of Psychology, University of Minnesota, Minneapolis, MN USA

**Keywords:** Ethnic-racial identity, Outgroup attitudes, Diversity attitudes, Adolescence, Intervention, The Identity Project

## Abstract

Outgroup and diversity attitudes are important components of intercultural understanding and well-being. Despite the potential of ethnic-racial identity development as a means to foster positive outgroup and diversity attitudes, little is known about its effectiveness in rapidly diversifying contexts such as Sweden. This pre-registered study filled this gap by examining if adolescents taking part in an intervention focused on ethnic-racial identity exploration, the Identity Project, also reported change in outgroup and diversity attitudes, and whether migration background, education type, and ethnic-racial identity development predicted such change. Twenty-three tenth-grade classes in Sweden (*N* = 509; *M*_*age*_ = 16.28; *SD*_*age*_ = 0.80; 66% female; 51% migration background) participated in the intervention and were assessed in four waves over a period of 26 weeks. Whereas ethnic-racial identity exploration and resolution increased for the intervention group, the adolescents reported no change in outgroup and diversity attitudes when compared to a control group. Increases in ethnic-racial identity exploration and resolution co-varied with increases in attitudes, but only at Time 3. The results do not provide support for the link between ethnic-racial identity development and positive outgroup and diversity attitudes, and challenge the notion of attitude change as a cascading effect of the Identity Project intervention in non-US sociocultural contexts. All aspects of the study were pre-registered on the Open Science Framework platform (https://osf.io/f5896).

## Introduction

Ethnic-racial identity development is an important developmental task for adolescents in culturally diverse settings (Yip, 2018). It involves many different processes, including how individuals explore the meaning and relevance of their ethnic, racial, and cultural backgrounds (Williams et al., 2020), as well as how they come to a sense of clarity or resolution about the roles these backgrounds play in their identity (Phinney et al., 1990). Ethnic-racial identity development has been linked to beneficial indicators of health, such as lessened depressive symptoms, greater academic achievement, and higher self-esteem (Rivas-Drake et al., 2014b), as well as positive attitudes about one’s own and other ethnic groups (Phinney, 2006). Given this evidence, interventions targeting adolescents’ ethnic-racial identity exploration has surfaced, such as the Identity Project, an eight-week school-based curriculum (Umaña-Taylor & Douglass, 2017). Research with the Identity Project has found evidence of its effects on ethnic-racial identity exploration and resolution (Umana-Taylor et al., 2018), and also positive relations with outgroup attitudes, or how individuals think about other people that they perceive to be part of different ethnic-racial groups (Juang et al., 2020). This initial evidence is limited, however, as outgroup attitudes were only assessed as a distal, downstream correlate of ethnic-racial identity processes, and relied on a single measure of outgroup attitudes that did not include individuals’ views concerning various cultural backgrounds, traditions, values, and identities within a society or community. Accordingly, the purpose of the present study was to investigate whether targeting ethnic-racial identity exploration can result in positive outgroup and diversity attitudes for adolescents in Sweden; a culturally diverse setting where demographics have changed rapidly over the last two decades (Statistics Sweden, 2023a). Furthermore, the study examined the associations between ethnic-racial identity development and change in outgroup attitudes, and whether adolescents’ migration background or type of educational program moderate such associations.

### Outgroup and Diversity Attitudes

The current study focuses on two aspects of attitudes: outgroup attitudes and diversity attitudes. *Outgroup attitudes* refer to an individual’s evaluations, feelings, and level of engagement with individuals or groups they perceive as different or external to their own social, ethnic, racial, or cultural group (Phinney, 1992). These attitudes include stereotypes, prejudices, biases, and feelings of affinity or animosity in regard to members of other groups. Outgroup attitudes play a significant role in intergroup interaction and trust (Hewstone et al., 2018), and can impact how individuals relate to those from different backgrounds or identities (Ülger et al., 2018). Positive outgroup attitudes have been linked to a sense of intergroup belonging and support, and are typically described as something individuals, schools, and societies should aspire towards (European Council on Tolerance and Reconciliation, 2023). Fostering outgroup attitudes, sometimes also referred to as intergroup attitudes, has been associated with more positive school experiences for adolescents (Graham et al., 2014), general well-being (Mendoza-Denton & Page-Gould, 2008), and intergroup contact (Rivas-Drake et al., 2019). As such, outgroup attitudes are important for ensuring healthy and positive relations for adolescents in any increasingly diverse society, such as Sweden, due to the role of attitudes in counteracting prejudice and forming non-negative orientations toward others (Dunn et al., 2009). The research field of outgroup attitudes and similar concepts is well-studied (Dovidio et al., 2018), but has long struggled with conceptualization (Ferrar, 1976), primarily regarding the term *tolerance* (i.e., tolerant attitudes toward others; Allport et al., 1954). The term, although widely used, can be understood as someone putting up with something they dislike, and also indicates that before one can tolerate something one has to be prejudiced against it (Rapp & Freitag, 2015). As such, a more value- or attitude-oriented terminology could be beneficial going forward, with a focus on how one responds to the existence of cultural diversity itself (Hjerm et al., 2020).

Diversity attitudes, the second central attitude concept in the current study, refer to an individual’s beliefs and inclinations concerning various cultural backgrounds, traditions, and identities within a society or community (UNESCO, 2005). These attitudes encompass how individuals perceive, appreciate, respect, accept, and engage with different cultures, and thus also their openness to engage with diversity (Pettigrew, 1998). Positive diversity attitudes play an important role in shaping intercultural interactions (Schachner et al., 2021), and likely does so by promoting inclusivity and fostering positive relationships among individuals from various different cultural backgrounds (Thijs & Verkuyten, 2013).

Outgroup and diversity attitudes are important during adolescence as this is a period of identity formation (Kroger et al., 2010), but also due to increased exploration and engagement with peers (Wölfer et al., 2016). Cognitive and social skills that develop during the period, such as mentalization and perspective-taking, also enhance one’s understanding of commonalities and differences between groups, leading to a broader understanding of societies and the groups within (Turner & Tajfel, 2004). Additionally, high schools tend to be larger and more culturally diverse than middle schools, thus providing greater exposure to a wider variety of people from different backgrounds during adolescence (Swedish National Agency for Education, 2022). Indeed, better intercultural relations and more opportunities to learn about cultural diversity in school have been found to be possible pathways to positive diversity attitudes as well as school adjustment (Schachner et al., 2016). In line with this, adolescents who perceive that their school provides opportunities to learn about topics such as ethnic and cultural heritage also report engaging in greater ethnic-racial identity exploration and commitment (Byrd & Legette, 2022). Furthermore, positive outgroup attitudes are instrumental in adolescents feeling included at school (Brown, 2019), but also require that schools establish prosocial and inclusive classroom norms that foster a favorable climate for all adolescents (Schwarzenthal et al., 2018).

There are multiple interventions focused on promoting positive outgroup and diversity attitudes, most of them delivered through the school setting (e.g., Durlak et al., 2011). A systematic review of 32 studies in which interventions were used to reduce prejudice and discrimination in children found a mixed picture: 40% of the interventions had positive effects on outgroup attitudes, 50% showed non-significant effects, and the last 10% showed adverse effects (Aboud et al., 2012). Another meta-analysis, containing 81 research reports and a total of 122 structured intervention-control programs aimed at promoting positive outgroup attitudes in adolescents via reductions in prejudiced attitudes, found that mean effect sizes were typically small (*d* = 0.30), indicating low to moderate intervention effects (Beelmann & Heinemann, 2014). A third meta-analytic review, with 50 studies, supported moderate intervention effects on outgroup attitudes through anti-bias programs in school, but found that these were most effective when delivered one-on-one and by researchers (Ülger et al., 2018). A commonality among these studies is that intervention programs typically revealed stronger effects in outgroup attitude change for adolescents from ethnic majority groups, but also that outgroup attitudes likely improve more in schools with ethnically balanced populations (van Zalk and Kerr, 2014), indicating that such interventions may have worked differently for different groups. One of the criticisms of school-based intervention work targeting outgroup attitude change is that it tends to be overtly practical: psychological theories and findings are rarely incorporated, and the impact on outgroup relations or attitudes is rarely evaluated systematically (Cameron & Turner, 2010). Such interventions also rarely focus on adolescents’ self-exploration but instead target group dynamics (Beelmann & Heinemann, 2014). One intervention grounded in decades of identity development research is the Identity Project, which focuses on adolescents’ identity exploration rather than external attitude change (Umaña-Taylor et al., 2018). Being given time and space to explore one’s ethnic-racial identity through interventions such as the Identity Project may, in fact, also be a pathway to positive outgroup and diversity attitudes (Juang et al., 2020).

### Ethnic-Racial Identity and Intervention

The Identity Project is a school-based intervention that originated in the US and consists of eight lesson plans targeting adolescent ethnic-racial identity exploration (Umaña-Taylor & Douglass, 2017). Throughout the sessions, adolescents explore their own ethnic and cultural backgrounds together with their classmates during regular school hours while simultaneously learning about topics such as social identity, between- and within-group differences, current and historical discrimination of minority groups, symbols, traditions, and more. The intervention focuses on active self-exploration and is highly inclusive, providing a way for students of all ethnic, racial, and cultural backgrounds to address and explore their ethnic-racial identity. In the original study conducted in the U.S., the Identity Project intervention stimulated increases in adolescents’ ethnic-racial identity exploration, measured at a 12-week post-test, which was linked to higher ethnic-racial identity resolution and better psychosocial adjustment at a 1-year post-intervention test, including positive changes in outgroup attitudes (Umaña-Taylor et al, 2018). Subsequently, the Identity Project has been implemented in several European school environments, where similar, but not identical, results on positive ethnic-racial identity development have been found in Italy (Ceccon et al., 2023), Germany (Juang et al., 2020), and Sweden (Abdullahi et al., 2024). In the German evaluation, cross-sectional support for ethnic-racial identity exploration was found in one of their cohorts, but not for resolution, as well as increased scores in critical consciousness and outgroup attitudes for the intervention group (Juang et al., 2020). In the Italian evaluation, positive longitudinal effects were found for ethnic-racial identity exploration processes, but not for resolution (Ceccon et al., 2023). In the Swedish evaluation, taking part in the Identity Project intervention led to increases in both ethnic-racial identity exploration and resolution, but not through the same stage-wise pattern as originally hypothesized (Abdullahi et al., 2024). While all activities and lesson goals of the original intervention were retained when adapted to the Swedish context, changes to the intervention included both surface and deep structure adaptations, such as the terminology that was used, the addition of discussions surrounding the immigrant/Swedish dichotomy, and an increased focus on regional identities (see Juang et al., 2023 for an extensive list of rationales and adaptations, and the OSF online material at https://osf.io/tpuxe for the eight-week intervention curriculum).

Although it has been suggested that ethnic-racial identity exploration and resolution have a cascading effect (the initiation of one leading to the initiation of the other) on outgroup attitudes (Umaña-Taylor & Douglass, 2017), little research has actually examined the question. One recent study examining the link between ethnic-racial identity and attitudes found that ethnic-racial identity resolution predicted self-esteem, which predicted outgroup attitudes (Wantchekon et al., 2023), indicating possible complex underlying mechanisms. Testing the potential effects of ethnic-racial identity exploration and resolution on outgroup and diversity attitudes is especially relevant, as individuals with a strong sense of identity are more likely to develop prodiversity attitudes over time (Erentaitė et al., 2019). This phenomenon may stem from how individuals who are more confident in their beliefs and values can explore their identity from a more secure position, allowing for more open-mindedness and acceptance of different perspectives (Phinney et al., 2007), facilitating life in culturally diverse settings. Conversely, individuals struggling with their identity development may be more likely to exhibit negative attitudes toward others, possibly because they may feel threatened by beliefs and values that differ from their own, and may seek to assert their own identity by rejecting those who are different (Tajfel & Turner, 2004). Taken together, it is possible that interventions targeting ethnic-racial identity development, such as the Identity Project, also directly affect outgroup and diversity attitudes. As adolescents are also encouraged to work together in smaller groups during several of the eight Identity Project sessions, intergroup contact may also increase among them. Intergroup contact, in turn, has been shown to promote reduced prejudice (Pettigrew & Tropp, 2006), positive attitudes toward outgroups, and relations, and increased intergroup cooperation (Wright et al., 2017).

When examining the possible relationships between an ethnic-racial identity intervention and attitudes, another important factor to consider is adolescents’ migration or non-migration backgrounds. Adolescents with a migration background are typically prompted to explore their ethnic-racial identity earlier in life (Syed & Azmitia, 2009), and do so at faster pace compared to adolescents from ethnic majorities (French et al., 2006). Whether this difference in exploration also impacts attitude development is not known, as no studies have examined if there are different developmental trajectories for outgroup and diversity attitudes among adolescents from different migration backgrounds when promoted through ethnic-racial identity development. Additionally, adolescents’ type of education (i.e., their choice of academic path during high school) may also be associated with their response to the intervention. The difference between theoretical programs (programs aimed at further academic studies) and more practically oriented ones (programs focused on craft skills) are socio-educational groupings that may impact the development of attitudes, and studies from the Swedish context have in fact shown that adolescents who study in practical high school programs tend to report less positive attitudes than those enrolled in theory-based high school programs do (Lundberg & Abdelzadeh, 2017). Thus, similar to how ethnic-racial identity tends to develop earlier and at a quicker pace for adolescents who are in the minority in their context, it is possible that outgroup and diversity attitudes also develop differently depending on social grouping factors such as adolescents’ migration background or type of education. Before the hypotheses based on these prior findings are presented, it is important to further acknowledge the sociocultural setting of the current study.

### The Swedish Sociocultural Context

Located in northern Europe and with a population of roughly 10 million, Sweden is an ethnoculturally diverse country with 26% of Swedes having at least one parent born outside Sweden (38% under 18 years; Statistics Sweden, 2023b). While changing demographics are the norm in most European countries due to forced displacements, globalization, and migration flows, Sweden has seen a rapid diversification of its society, with migration-based diversity two decades ago being approximately half of what it is today (Statistics Sweden, 2023a). The geographical locations from which people have immigrated have changed throughout history, and currently, the three most common countries of birth outside of Sweden are Syria, Iraq, and Finland. Sweden often scores very high on the Multicultural Policy Index (2021), indicating general support for policies toward immigrant minorities, and is often recognized for its strong commitment to social welfare and equality (Berggren & Trägårdh, 2015). At the same time, research also shows that ethnic segregation is increasing in the country (Hedström, 2019), and similar to many European countries, anti-immigrant attitudes have been (Valdez, 2014), and again is on the rise (European Social Survey, 2023). Adolescents with migration backgrounds commonly describe that they experience discrimination and racism (City of Gothenburg, 2023), as well as feelings of not fitting into the dichotomous, narrow frame of being considered a “Swede” (Gyberg et al., 2018). As such, adolescents in Sweden form a highly diverse group, yet many share the everyday experience of having a migration background and belonging to one or more minoritized ethnic groups. This shared experience among adolescents of being minoritized, or navigating multiple cultural backgrounds, has been associated with increased perceptions of discrimination and adverse health effects (Mock-Munõz de Luna et al., 2019), such as lower levels of self-esteem and depressive symptoms (Bayram Özdemir et al., 2021b).

Many adolescents in Sweden, both with and without migration backgrounds, are as such tasked with exploring their ethnic-racial identities in an ethnically diverse setting, prompting complex acculturation processes for many of them (the task of incorporating, or not incorporating one’s heritage backgrounds into a new community; Svensson & Syed, 2019). While prior research suggests that the development of positive outgroup attitudes differs among adolescents from migration and non-migration backgrounds (Lundberg & Abdelzadeh, 2017), the majority of adolescents in Sweden seemingly regard themselves as harboring positive interpersonal attitudes (Björklund & Dahlberg, 2017), and these attitudes tend to slowly increase throughout the span of adolescence. These sociocultural dynamics and the potential for preventive work make Sweden an important context to study in regard to both ethnic-racial identity development and the fostering of outgroup and diversity attitudes.

## Current Study

Despite the potential of ethnic-racial identity development as a means to foster positive outgroup and diversity attitudes amongst adolescents, little is known about its effectiveness, especially in rapidly diversifying contexts such as Sweden. This pre-registered study (https://osf.io/f5896) addresses this gap in knowledge through an intervention and wait-list control design with four measurements spanning 26 weeks, T1-T4. Half of the participating adolescents received the Identity Project intervention after baseline, T1 (intervention group), and the other half received the intervention before the final measurement, T4 (wait-list control group). Based on prior research on ethnic-racial identity development, attitude development, group differences in trajectories, as well as existing Identity Project intervention evaluations from the US, Germany, and Italy, four hypotheses and one exploratory analysis were formulated and tested. Positive mean level change in outgroup and diversity attitudes were expected between T1 and T4 for adolescents in the intervention group (Hypothesis 1a). Stable mean levels of outgroup and diversity attitudes were expected for adolescents assigned to the wait-list control group between T1 and T3 (Hypothesis 1b). An un-specified interaction effect between migration background status and change in outgroup and diversity attitudes was expected for the intervention group between T1 and T4 as this status was also expected to impact ethnic-racial identity development (Hypothesis 2a). It was explored whether adolescents’ types of education program (theoretical or practically oriented) predicted change in outgroup and diversity attitudes for the intervention group between T1 and T4 (Exploration 2b). Lastly, positive change in ethnic-racial identity exploration and resolution between T1 and T4 were expected to be associated with positive change in outgroup and diversity attitudes for adolescents, regardless of intervention or control group status (Hypothesis 3).

## Method

### Participants

During the last quarter of 2021 and the first quarter of 2022, 22 tenth-grade classes from four high schools were recruited and took part in four waves of survey measurements, T1 through T4. A total of 509 adolescents participated in the study (*Age*_*range*_ = 15–19; *M*_*age*_ = 16.28; *SD*_*age*_ = 0.80; 66% female; 51% migration background; 66% theoretical education). Half of the classes from each school were randomly assigned to the experiment group, and the other half to the wait-list control. Adolescent migration background status was coded based on self-reports of one’s own and one’s parents’ birth country. In line with how the Swedish National Statistics Bureau categorizes backgrounds, adolescents with two parents born abroad, regardless of birthplace, were coded as having migration backgrounds. Adolescents with one or more parents born in Sweden, regardless of birthplace, were coded as having a non-migration background. If at least one parent’s birth country was missing and the adolescent was born in Sweden, the adolescent was coded as having a non-migration background. If at least one parent’s birth country was missing and the adolescent was born abroad, the adolescent was coded as having a migration background (Statistics Sweden, 2002). The adolescents’ type of education was classified as attending either a theoretical or a practical education program. Theoretical education programs included three-year programs in the natural sciences, economics, or the social sciences, while practical education programs included one to three years in childcare, caretaking, craftsmanship, agriculture, or individually adapted programs. The intervention and control groups did not differ in terms of migration background (χ^2^(1) = *1.95, p* = 0.21) or type of education (χ^2^(1) = 2.26 *p* = *0*.13), and subsequent participant retention rates were strong over time (T2 = 84.7%; T3 = 84.9%; T4 = 80.0%). While few adolescents missed more than one measurement, the overall attrition rate of the sample was moderate-to-high, with 39% of adolescents having missing data at least one subsequent time point compared to their baseline assessment. Differentiating between random and non-random patterns, only one discernible pattern of missingness was found; a cumulative increase in missing data for all variables between consecutive time points (e.g. diversity attitudes: T1_miss_ = 16.1%; T1_miss_ = 20.4%; T1_miss_ = 23.4%; T1_miss_ = 26.3%). Attrition across time and drop-out were not associated with participants gender (*p* = 0.13), migration background (*p* = 0.73), T1 diversity and outgroup attitudes (*p* = 0.13; *p* = 0.09), or T1 ethnic-racial identity development (*p* = 0.20; *p* = 0.32; *p* = 0.48), but was associated with participants type of education (χ^2^(1) = 13.1, *p* = < 0.01), meaning that adolescents in classrooms of practical education type was more likely to drop out or miss one or more measurements. Adolescents with missing data for any reason other than lacking or canceling participation were included in the dataset using a Full Information Maximum Likelihood (FIML) approach to account for missingness, meaning that some adolescents were missing from the baseline but were still included in the sample.

### Procedure

High school principals at schools with ethnically diverse classrooms in the region of Västra Götaland, Sweden, were given information regarding the Identity Project (dubbed “LIKE” in Swedish, an acronym for “learning about identity, culture, and ethnicity”) via e-mail and then via a phone call. In order to receive sociodemographic information at a school level, the Swedish National Agency for Education’s online information system, SIRIS, was used (siris.skolverket.se). Meetings with interested schools were scheduled to allow principals and teachers to ask clarifying questions about the intervention program, after which the schools were invited to participate. Students from the participating classes were informed about the upcoming intervention by visiting research team members and their respective teachers and also received the same information in written form. All participating schools agreed to implement the intervention sessions as part of their ordinary education (most often in the form of sessions delivered during a social sciences class) rather than adding extra hours to the students’ schedules.

On four occasions, T1-T4, the adolescents answered questionnaires provided in Swedish via an online Qualtrics platform. Adolescents in the experiment group received the Identity Project intervention between T1 (baseline) and T2 (+10 weeks), while the wait-list control group received the intervention between T3 (+16 weeks) and T4 (+26 weeks). The last measurement, T4, was as such not a true control group design, something addressed further under the heading “analytic strategy”. Participating adolescents could complete the questionnaires on their phones or their school-assigned computers, but had to remain in the classroom. They were offered a snack while filling out the forms but received no other compensation. Before the measurements, a professional translated the questionnaires into Swedish versions when such were not available. In order to keep information from being lost or added to any of the items, the translated versions were back-translated into English and compared to the authors’ original scales. After these initial translations, a pilot measurement was conducted with 30 high school students who were encouraged to mark questions or phrasings they had difficulty understanding and to give the facilitators any other feedback regarding the questionnaires. Minor linguistic alterations were made to the translated items based on these re-translations and pilot group feedback. In order to assist with understanding some of the items in the questionnaire, a word list with explanations was handed out to all adolescents during all measurements. During the measurements, one to three moderators (and often one teacher) were present in the classroom to help answer questions or provide language-based assistance. All moderators were trained clinical psychologists and actively took part of the research program. In all but one class, moderators were teamed up so that both majority and minoritized ethnic backgrounds were represented.

### Measures

#### Outgroup attitudes

Measured using the Other-Group Orientation subscale of the Multigroup Ethnic Identity Measure, MEIM-OGO (Phinney, 1992). The subscale consists of six items measuring adolescents’ general attitudes and willingness to spend time with individuals with ethnic-racial and cultural backgrounds that differ from their own, e.g. “I like meeting and getting to know people from cultural groups other than my own”. The items are answered on a four-point Likert scale ranging from 1 (Strongly disagree) to 4 (Strongly agree). Internal consistency of the scale was slightly below adequate (α_T1_ = 0.57; α_T2_ = 0.54; α_T3_ = 0.60; α_T4_ = 0.55).

#### Diversity attitudes

Measured on three scales: acceptance of diversity, respect for diversity, and appreciation of diversity (Hjerm et al., (2020). While the term tolerance is used by the scales’ originators, the items do not capture values or attitudes regarding specific outgroups, ideas, or behaviors; therefore, the broader term *diversity attitudes* is used throughout this paper instead. Aggregated, the three scales consist of eight items: three concerning acceptance of diversity, two concerning respect for diversity, and three concerning appreciation of diversity. Items include “It is important that people have the freedom to live their life as they choose,” “I respect other people’s beliefs and opinions,” and “Society benefits from a diversity of traditions and lifestyles”. The items are answered on a five-point Likert scale ranging from 1 (Strongly disagree) to 5 (Strongly agree). Internal consistency of the scale was excellent (α_T1_ = 0.92; α_T2_ = 0.90; α_T3_ = 0.91; α_T4_ = 0.93).

#### Ethnic-racial identity exploration

Measured using the exploration subscales of the Ethnic Identity Scale – Brief (EIS-B) (Douglass & Umaña-Taylor, 2015) and the Multigroup Ethnic Identity Measure (MEIM) (Phinney, J., 1992; Roberts et al., 1999). The EIS-B exploration subscale includes three items measured using a four-point Likert scale ranging from 1 (Does not describe me at all) to 4 (Describes me very well). The MEIM exploration subscale includes five items on a four-point Likert scale ranging from 1 (Strongly disagree) to 4 (Strongly agree). Two subscales were used because the items in the EIS-B focus on participatory aspects of ethnic-racial identity exploration, e.g. “I have attended events that have helped me learn more about my ethnicity,” while MEIM focuses more on an exploratory search aspect, e.g. “I think a lot about how my life will be affected by my ethnic group membership”. Internal consistency of the EIS-B was very good (α_T1_ = 0.80; α_T2_ = 0.82; α_T3_ = 0.85; α_T4_ = 0.85). Internal consistency of the MEIM was good (α_T1_ = 0.69; α_T2_ = 0.76; α_T3_ = 0.79; α_T4_ = 0.80).

#### Ethnic-racial identity resolution

Measured using the resolution subscale of the Ethnic Identity Scale – Brief (EIS-B) (Douglass & Umaña-Taylor, 2015). The subscale includes three items, e.g. “I have a clear sense of what my ethnicity means to me,” and is measured using a four-point Likert scale ranging from 1 (Does not describe me at all) to 4 (Describes me very well). Internal consistency of the EIS-B was very good (α_T1_ = 0.80; α_T2_ = 0.82; α_T3_ = 0.85; α_T4_ = 0.85).

### Analytic Strategy

All reported analyses were pre-registered (https://osf.io/f5896) unless otherwise specified. Descriptive statistics and plotted data were examined to screen for apparent patterns and outliers. Extreme outliers and careless responders were identified using least median square (LMS) descriptives, and careless responding was analyzed using the R-package *careless* (Yentes & Wilhelm, 2021). In order to detect possible leverage from outliers in the data (which may impact slopes and intercepts), robust model-based latent factor and residual analysis (RFRA) were employed during the growth modeling process (Tong & Zhang, 2017). Baseline characteristics of the sample were examined to assess potential differences between groups and compared using the Wilcoxon signed-rank test, one-way ANOVAs, and independent t-tests. Attrition and drop-out were measured by creating binary variables for adolescents with one or more missing data points, followed by chi-square and Wilcoxon tests to assess the differences. Intraclass correlation coefficients (ICC) were calculated to assess variability at three different levels (waves in individuals; in the classroom; in school). Latent growth curve models (LGCM) in the *lavaan* package of R-statistics (Rosseel, 2012) were used to assess mean-level change in all dependent and independent variables. Latent growth parameters (intercepts, slopes), as well as means and standard errors for linear and quadratic unconditional models, were examined and used to test the significance of attitude growth in a sequential fashion, starting with linear models followed by quadratic models.

The wait-list control design presented some challenges for comparing the intervention and control groups across time, given that the control group also received the intervention between T3 and T4, and thus the T4 assessment for the control group is post-intervention. This was handled in two ways. First, a separate data set was created where all scores at T4 for the control group were coded as missing. Because the analyses used FIML with all available data, the control groups’ growth up until T4 was created in the model and based on the groups’ trajectory between T1 and T3. This was deemed a plausible solution as the mechanism of missingness is known—because they were in the control group—and the reason for the missingness (intervention vs. control group) is included as a covariate in the model (Curran et al., 2010). As a non-registered test of robustness, analyses were also run on the full data set (i.e., without setting T4 in the control group to missing) with a time-varying factor that accounted for the change in intervention/control status for the control group between T3 and T4. These analyses did not yield any substantive differences from the previous set of analyses, and thus only the simpler analyses with T4 for the control group treated as missing are presented in this manuscript. While pre-registered, shifting the model intercepts between timepoints to assess how baseline levels affected outcome variables over time revealed no group differences and were left out of the manuscript.

Fit statistics such as the sample size-adjusted Bayesian information criterion (ssBIC) and likelihood ratio testing based on -2LL values for the respective models were assessed to determine which models to keep. Random effects (level 2 residuals) were fixed to zero when the procedure did not cause any loss of model fit in order to gain more degrees of freedom in the analyses. Participants’ assignment to the intervention or control was entered as binary predictors of intercepts and slopes for all models but one (Hypothesis 3 testing). Type of education (theoretical or practical) and migration background status (yes/no) were also entered as binary predictors of intercepts and slopes for their respective hypotheses. Multivariate latent growth curve models were run to detect associations between ethnic-racial identity development and attitude measures and to gauge whether these functioned as continuous predictors of one another. The parsimonious models were always prioritized and used. In order to understand other possible longitudinal path effects within the growth model, fixed intercepts were shifted between time points to examine different points at which ethnic-racial identity was associated with a change in attitudes. A Full Information Maximum Likelihood (FIML) approach was employed throughout the latent growth curve modeling with robust standard errors to account for missing data.

Inferences were drawn for all parametric analyses at *p* < 0.05. Rank-order stability analyses were included in the analyses as part of the latent growth curve models, which include the consistency of individuals’ relative positions within a group over time. Two unregistered exploratory analyses were added in the later stages of data analysis: latent growth curve models for assessing possible interaction effects between migration background status/type of education and intervention/control group condition. All data analyses and models were run and fit in RStudio Build 576 (R Core Team, 2021; R Studio Team, 2020). Sample size determination, exclusion criteria, all manipulations, all measures, and all analyses in the study are reported. The following growth-parameter notations are used throughout the manuscript: *I* to indicate intercepts calculated within the model, *S*_*L*_ to indicate linear slopes, and *S*_*Q*_ to indicate quadratic slopes.

## Results

### Preliminary Analyses

Several preliminary analyses were performed to assess the nature and quality of the dataset. In order to assess the similarity of observations within the sample, multiple levels of Intraclass Correlation Coefficients (ICC) were performed, treated as time-points nested within individuals, nested within classrooms, nested within schools. ICC for time-points within individuals revealed sufficient variation in adolescents’ scores on attitudes and identity development over time (*r*_outgroup_ = 0.50; *r*_diversity_ = 0.40; *r*_explorationEIS_ = 0.43; *r*_explorationMEIM_ = 0.48; *r*_resolution_ = 0.48). Classroom variability accounted for very little of the variation (*r*_outgroup_ = 0.01; *r*_diversity_ = 0.01; *r*_explorationEIS_ = 0.02; *r*_explorationMEIM_ = 0.06; *r*_resolution_ = 0.03), and similar low levels of variability were observed at the school level (*r*_outgroup_ = 0.09; *r*_diversity_ = 0.02; *r*_explorationEIS_ = 0.05; *r*_explorationMEIM_ = 0.09; *r*_resolution_ = 0.11). Neither of these coefficients was deemed large enough to significantly impact the modeling and were thus not included further. Longstring boxplots were used to visually gauge outliers in the data (adolescents with straight-lined responses, e.g. “4, 4, 4, 4, 4”), and careless responses were analyzed using the intra-individual response variability function (*IRV* = 0.88–1.61). These two analyses indicated a spread in the standard deviation of responses across the dataset, and no major amounts of straight-lined responses among adolescents. To summarize, the dataset included some expected, but no excessive or data-compromising, amounts of inconsistencies or careless responses.

Correlations between the three diversity attitude measures (acceptance of, respect for, and appreciation of diversity) ranged between strong correlations and very strong correlations within time points (*r* = 0.68–0.81), indicating that they measure the same global construct (diversity attitudes). Although aggregating these three scales into one (diversity attitudes) was reasonable due to high correlations between the three, the strategy provided no new information in the hypothesis testing. As a test of robustness, aggregated diversity attitude scores were ran throughout and are included in the online supplementary material (https://osf.io/t8km5). Correlations between outgroup attitudes and the three diversity attitude measures ranged from very weak to weak (*r*_range_ = 0.11–0.35), indicating that outgroup attitudes is a separate construct from diversity attitudes. It is also important to point out that auto-correlations for all attitude measures were sometimes low (*r*_range_ = 0.28–0.61), and while this could impact stability, such correlations are also rather common in psychosocial research on adolescents’ attitudes, a time period were attitude scores are likely to fluctuate (see for example Bayram Özdemir et al., 2021a; Bohman & Miklikowska, 2021; Munniksma et al., 2015). Means, standard deviations, and bivariate correlations of all main measurements at all four-time points are presented in Table 1 (for descriptive data separated by groups, see https://osf.io/ejbt8).

**Table 1:** Descriptive statistics and bivariate correlations for all measures used at T1, T2, T3, and T4

Variable	*M*	*SD*	1	2	3	4	5	6	7	8	9	10	11	12	13	14	15	16	17	18	19	20	21	22	23	24	25	26	27
1. ogo_t1	3.33	0.49																											
2. ac_t1	4.34	0.94	0.23																										
3. re_t1	4.07	0.91	0.11	0.76																									
4. ap_t1	3.87	0.89	0.29	0.75	0.73																								
5. eisE_t1	2.40	0.86	0.06	0.04	0.04	0.06																							
6. eisR_t1	3.21	0.66	0.09	0.09	0.14	0.16	0.47																						
7. meim_t1	2.60	0.65	0.05	0.02	0.04	0.07	0.44	0.28																					
8. ogo_t2	3.27	0.48	0.58	0.11	0.06	0.13	−0.01	0.00	0.05																				
9. ac_t2	4.34	0.84	0.24	0.43	0.28	0.27	−0.03	−0.01	−0.01	0.28																			
10. re_t2	4.04	0.86	0.15	0.27	0.36	0.25	−0.00	0.07	0.05	0.21	0.71																		
11. ap_t2	3.83	0.82	0.29	0.29	0.28	0.36	0.02	0.04	0.03	0.33	0.70	0.68																	
12. eisE_t2	2.67	0.78	0.08	0.09	0.04	0.15	0.49	0.31	0.38	0.10	0.01	0.02	0.08																
13. eisR_t2	3.26	0.61	0.15	0.11	0.13	0.15	0.28	0.62	0.25	0.11	0.08	0.14	0.11	0.49															
14. meim_t2	2.65	0.67	0.01	0.04	−0.00	0.02	0.36	0.23	0.48	−0.01	0.03	0.03	0.02	0.45	0.27														
15. ogo_t3	3.23	0.50	0.48	0.18	0.15	0.20	0.06	0.06	0.06	0.58	0.28	0.22	0.30	0.17	0.13	0.07													
16. ac_t3	4.26	0.88	0.18	0.41	0.26	0.27	−0.09	−0.06	−0.00	0.18	0.53	0.39	0.37	−0.08	−0.01	0.00	0.27												
17. re_t3	4.04	0.90	0.13	0.32	0.35	0.26	0.00	0.07	0.04	0.08	0.40	0.50	0.34	−0.05	0.05	0.00	0.19	0.74											
18. ap_t3	3.92	0.82	0.18	0.22	0.22	0.32	−0.02	0.04	0.06	0.17	0.34	0.40	0.45	0.00	0.04	0.02	0.28	0.68	0.69										
19. eisE_t3	2.74	0.81	0.14	0.12	0.06	0.12	0.42	0.28	0.39	0.05	0.07	0.10	0.18	0.61	0.29	0.46	0.12	0.08	0.13	0.15									
20. eisR_t3	3.25	0.69	0.20	0.19	0.15	0.18	0.29	0.47	0.30	0.08	0.07	0.15	0.14	0.35	0.65	0.29	0.20	0.19	0.27	0.22	0.54								
21. meim_t3	2.76	0.68	0.10	0.03	0.06	0.08	0.43	0.31	0.50	0.13	0.01	0.07	0.13	0.41	0.31	0.59	0.13	0.02	0.08	0.13	0.50	0.33							
22. ogo_t4	3.19	0.50	0.45	0.17	0.13	0.19	−0.04	0.00	0.00	0.53	0.24	0.14	0.25	0.05	0.05	−0.06	0.61	0.22	0.15	0.23	0.11	0.12	0.05						
23. ac_t4	4.19	0.96	0.21	0.36	0.21	0.27	−0.04	0.01	−0.03	0.18	0.46	0.27	0.23	0.07	0.13	−0.02	0.27	0.58	0.44	0.39	0.07	0.09	0.03	0.34					
24. re_t4	4.00	0.91	0.10	0.26	0.23	0.23	0.01	0.11	0.02	0.17	0.25	0.36	0.25	0.11	0.15	−0.01	0.24	0.41	0.47	0.43	0.09	0.14	0.07	0.25	0.78				
25. ap_t4	3.88	0.87	0.22	0.25	0.16	0.30	−0.01	0.08	0.09	0.21	0.25	0.24	0.38	0.09	0.08	0.04	0.26	0.39	0.41	0.50	0.15	0.08	0.16	0.35	0.76	0.81			
26. eisE_t4	2.84	0.79	0.06	0.00	−0.01	0.00	0.43	0.24	0.42	0.09	0.03	0.02	0.10	0.49	0.25	0.54	0.10	0.06	0.07	0.10	0.52	0.31	0.46	0.12	0.04	0.07	0.10		
27. eisR_t4	3.32	0.64	0.12	0.07	0.02	0.10	0.29	0.43	0.27	0.06	0.05	0.07	0.13	0.27	0.55	0.22	0.16	0.22	0.26	0.23	0.36	0.56	0.26	0.10	0.18	0.19	0.15	0.51	
28. meim_t4	2.78	0.67	0.04	0.02	0.00	0.05	0.36	0.37	0.48	0.07	0.05	0.14	0.12	0.37	0.28	0.50	0.06	−0.04	0.05	0.02	0.35	0.30	0.54	0.05	0.07	0.14	0.18	0.42	0.26

M and SD are used to represent mean and standard deviation. All correlations were mapped using Pearson’s correlation coefficient. N_(range)_ = 313–454. *ogo* outgroup attitudes, *ac* acceptance of diversity, *re* respect for diversity, *ap* appreciation of diversity, *eisE* ethnic-racial identity exploration using EIS scale, *eisR* ethnic-racial identity resolution using EIS scale, *meim* ethnic-racial identity exploration using MEIM scale. Measure range for MEIM and OGO was Likert 1–4. The range of all other measures was Likert 1–5

Visual inspection of plotted raw data and histograms suggested linear change patterns and a positive skew for all four attitude measures (outgroup attitudes, acceptance of diversity, respect for diversity, and appreciation of diversity). Furthermore, converting and plotting data in long form revealed generally high scores on all attitude measures, indicating that the potential for positive change in both outgroup and diversity attitudes may be somewhat limited in the data due to ceiling effects, but also that statistical regressions toward the mean may have exaggerated any negative change in outgroup and diversity attitudes. Several growth models conditioned for intervention or control group belonging were fitted to gauge intervention effects on outgroup and diversity attitude change. All fit indices showed strong model fit for linear growth models regarding outgroup and diversity attitudes (*CFI*_outgroup_ = 0.99; *CFI*_acceptance_ = 0.97; *CFI*_respect_ = 0.99; *CFI*_appreciation_ = 0.99). For a complete list of model fit statistics, see https://osf.io/5ymf3. Comparative analyses showed that, for all four attitude measures, quadratic growth models did not fit better than linear models (χ2_outgroup_(3) = 8.85, *p* =0.07 *CFI*_*diff*_ = −0.01; χ2_acceptance_(3) = 7.68, *p* = 0.10 *CFI*_*diff*_ = −0.02; χ2_respect_ (3) = 5.96, *p* = 0.20 *CFI*_*diff*_ = −0.02; χ2_appreciation_(3) = 2.88, *p* = 0.58 *CFI*_*diff*_ = < −0.001), thus, the linear, parsimonious models were chosen and used in further analyses.

In order to assess possible baseline differences impacting the hypothesis testing, several comparison tests were run. Wilxocon signed-rank test indicated that attitude scores were not different at baseline when grouped by control or experiment group (*p*_diversity_ = 0.54; *p*_outgroup_ = 0.81) or migration background (*p*_diversity_ = 0.70; *p*_outgroup_ = 0.42). Outgroup attitudes differed at baseline when grouped by type of education (*p*_diversity_ = 0.27; *p*_outgroup_ = < 0.01). One-way ANOVAs revealed no differences in any attitudes when grouped by gender (*p*_diversity_ = 0.15; *p*_outgroup_ = 0.34), but found differences in outgroup attitudes when grouped by school (*p*_diversity_ = 0.72; *p*_outgroup_ = < 0.01), and classroom (*p*_diversity_ = 0.59; *p*_outgroup_ = < 0.01). Independent sample t-tests revealed some differences in terms of ethnic-racial identity development at baseline when grouped by control or experiment group (*p*_explorationEIS_ = < 0.01; *p*_*e*xplorationMEIM_ = 0.12; *p*_resolution_ = 0.72) and migration background (*p*_*e*xplorationEIS_ = < 0.01; *p*_*ex*plorationMEIM_ = < 0.01; *p*_resolution_ = < 0.01), but not when grouped by type of education (*p*_explorationEIS_ = 0.47; *p*_explorationMEIM_ = 0.69; *p*_resolution_ = 0.10). One-way ANOVAs revealed baseline differences in ethnic-racial identity development when grouped by gender (*p*_explorationEIS_ = 0.66; *p*_explorationMEIM_ = 0.88; *p*_resolution_ = 0.03), school (*p*_explorationEIS_ = < 0.01; *p*_explorationMEIM_ = < 0.01; *p*_resolution_ = < 0.01), and classroom (*p*_explorationEIS_ = < 0.01; *p*_explorationMEIM_ = < 0.01; *p*_resolution_ = < 0.01). Pairwise difference analyses using Tukey’s post hoc tests revealed that school and classroom differences at baseline were only significant for schools and classrooms in different cities, not for schools or classrooms within the same city. All the significant differences above were accounted for in the following latent growth curve analyses.

### Intervention Effect on Outgroup and Diversity Attitudes

There were no differences for outgroup or diversity attitude growth between the intervention and control group, failing to support Hypothesis 1a: that intervention group participation would predict positive outgroup and diversity attitude change when compared to a control group (*S*_*L*_*(diff)*_outgroup_ = −0.000, *p* = 0.88); *S*_*L*_*(diff)*_acceptance_ = 0.004, *p* = 0.38; *S*_*L*_*(diff)*_respect_ = 0.003, *p* = 0.51; *S*_*L*_*(control)*_appreciation_ = 0.000, *p* = 0.98). Although there were no group differences in the slope, there were group differences in the intercept (*I(diff)*_outgroup_ = 0.12, *SE* = 0.04, *p* = 0.004), indicating that the control group scored slightly higher than the intervention group on outgroup attitudes at baseline (for a model example, see https://osf.io/8xuam).

**Fig. 1:**
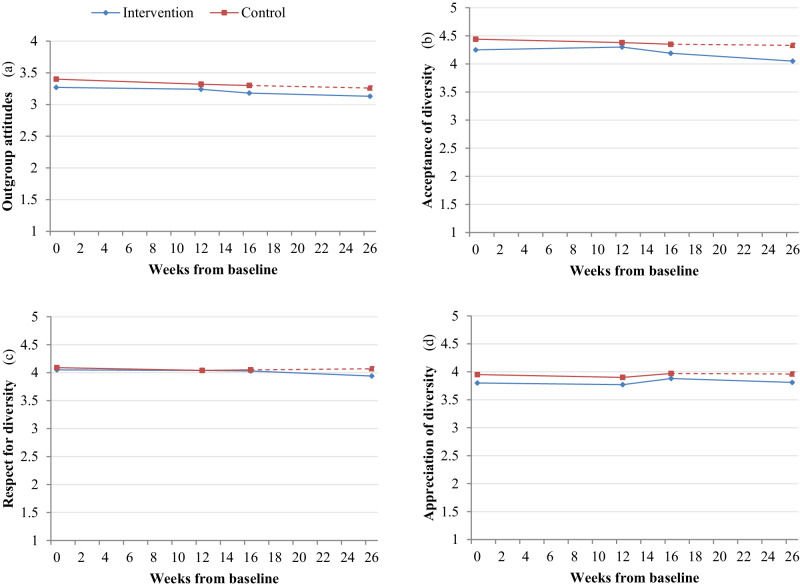
*Outgroup and diversity attitude growth for intervention and control groups, from baseline, T1 through T4*. Note. *N* = 509. *M*_age_ = 16.28; *SD*_age_ = 0.80. 66% female. 51% migration background. T1 measurement occurred at 0 weeks, T2 at +12 weeks, T3 at +16 weeks, and T4 at +26 weeks from baseline. Please see Table 2 for standard errors of trajectories

Although the intervention and control group differences were not significant in the full model, subsequent exploratory analyses examined outgroup and diversity attitude trajectories separately for the intervention and control groups (Table 2). Individual values for the intervention group revealed minimal but declining effects for outgroup attitudes (*S*_*L*_ = −0.006, *SE* = 0.002) and acceptance of diversity (*S*_*L*_ = −0.009, *SE* = 0.003). No group-level positive effect on slopes for outgroup attitudes or diversity subscales was found for adolescents in the intervention group. Individual values for the control group’s scores on outgroup and diversity attitudes did not generate significant changes for slopes in either direction (*S*_*L*_*(control)*_outgroup_ = 0.000, *SE* = 0.000; *S*_*L*_*(control)*_acceptance_ = 0.004, *SE* = 0.004; *S*_*L*_*(control)*_respect_ = 0.003, *SE* = 0.004; *S*_*L*_*(control)*_appreciation_ = 0.000, *SE* = 0.004), supporting Hypothesis 1b: Scores on outgroup and diversity attitudes remain stable between T1-T3 for adolescents assigned to the wait-list control group (Fig. 1).

**Table 2:** Individual growth parameters and standard errors for latent growth curve models – attitudes

Fixed effects, estimates	Intercept (*I*)	*SE*	Linear slope (*S*_*L*_)	*SE*
Outgroup Attitudes				
Unconditional	3.32	0.02	−0.006***	0.001
~ Control group	3.39	0.04	0.000	0.002
~ Intervention group	3.27	0.03	−0.006***	0.002
~ Migration background	3.34	0.05	−0.003	0.002
~ Non-migration background	3.35	0.04	−0.004*	0.002
~ Theoretical education program	3.40	0.03	−0.006***	0.001
~ Practical education program	3.19	0.05	0.002	0.002
Acceptance of diversity				
Unconditional	4.37	0.04	−0.007***	0.002
~ Control group	4.44	0.08	0.004	0.004
~ Intervention group	4.31	0.06	−0.009**	0.003
~ Migration background	4.27	0.08	−0.001	0.004
~ Non-migration background	4.48	0.05	−0.006*	0.003
~ Theoretical education program	4.38	0.05	−0.003	0.002
~ Practical education program	4.35	0.09	−0.012**	0.005
Respect for diversity				
Unconditional	4.07	0.04	−0.003	0.002
~ Control group	4.08	0.08	0.003	0.004
~ Intervention group	4.06	0.06	−0.004	0.003
~ Migration background	4.04	0.08	0.001	0.004
~ Non-migration background	4.09	0.05	−0.004	0.003
~ Theoretical education program	4.08	0.05	−0.000	0.002
~ Practical education program	4.05	0.09	−0.008	0.005
Appreciation of diversity				
Unconditional	3.86	0.04	0.000	0.002
~ Control group	3.94	0.08	0.000	0.004
~ Intervention group	3.80	0.06	0.000	0.003
~ Migration background	3.83	0.08	0.002	0.004
~ Non-migration background	3.90	0.05	−0.000	0.003
~ Theoretical education program	3.92	0.05	0.002	0.002
~ Practical education program	3.77	0.08	−0.006	0.004

All conditions were run T1-T4 with the T4 scores for the control group being treated as missing in order to account for when they received the intervention. For migration background and type of education, intervention/control group status was included as a predictor of growth coefficients. Time = 26 weeks from baseline to last measurement

*p < 0.05. **p < 0.01. ***p < 0.001

### Migration Background and Type of Education as Predictors of Change in Outgroup and Diversity Attitudes

**Fig. 2:**
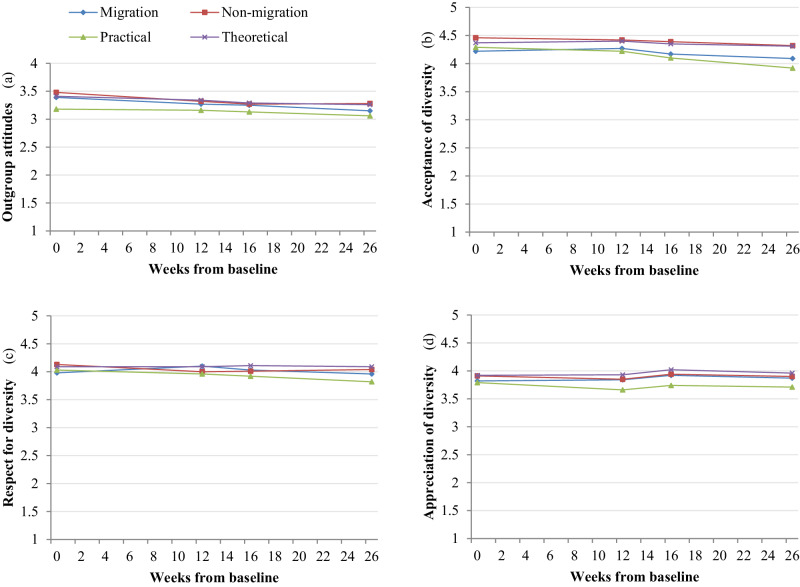
*Outgroup and diversity attitude growth for migration and education groupings, from baseline, T1 through T4. Intervention and control group status was included as a predictor of growth coefficients*. Note. *N* = 509. *M*_age_ = 16.28; *SD*_age_ = 0.80. 66% female. 51% migration background. T1 measurement occurred at 0 weeks, T2 at +12 weeks, T3 at +16 weeks, and T4 at +26 weeks from baseline. Please see Table 2 for standard errors of trajectories

Migration background was not a significant predictor of either outgroup or diversity attitudes (*S*_*L*_*(diff)*_outgroup_ = −0.003, *p* = 0.15); *S*_*L*_*(diff)*_acceptance_ = 0.001, *p* = 0.84; *S*_*L*_*(diff)*_respect_ = 0.001, *p* = 0.73; *S*_*L*_*(control)*_appreciation_ = 0.002, *p* = 0.69), failing to support Hypothesis 2a: that migration background status would predict change in outgroup and diversity attitudes for those in the intervention group. However, non-migration background status predicted higher baseline scores for acceptance of diversity (*I(diff)*_*acceptance*_ = −0.020, *SE* = 0.008, *p* = 0.013) but not for other attitude measures. Although group differences were not significant, further analyses examined individual attitude trajectories separately for the migration and non-migration background groups. Whereas there was no change among adolescents with a migration background, those with a non-migration background showed a decrease in outgroup attitudes (*S*_*L*_ = −0.004, *SE* = 0.002) as well as acceptance of diversity (*S*_*L*_ = −0.006, *SE* = 0.003). No positive effects in slopes for outgroup and diversity attitudes were found for adolescents in either of these groups (Fig. 2).

Primary testing of the exploratory analysis 2b, that adolescents’ type of education program would predict change in outgroup and diversity attitudes for those in the intervention group, found one difference between the groups in terms of acceptance of diversity (*S*_*L*_*(diff)*_acceptance_ = −0.012, *SE* = 0.005, *p* = 0.007). There was also one minor but significant difference in intercepts between adolescents from theoretical education programs and practical education programs in terms of outgroup attitudes at baseline (*I(diff)*_outgroup_ = 0.21, *SE* = 0.05, *p* = <0.001). These results show that adolescents in a theoretical education program scored higher on outgroup attitudes at baseline and had steeper negative change in acceptance of diversity. Attitude trajectories within the education group types were individually examined next. Results show that attending a theoretical education program predicted a small but declining slope effect in outgroup attitudes (Fig. 1a; *S*_*L*_ = −0.006, *SE* = 0.001), while attending a practical education program predicted similar small but declining slope effects for acceptance of diversity (Fig. 1b; *S*_*L*_ = −0.012, *SE* = 0.005). With significant but small differences in slopes for acceptance of diversity but no other attitude measures, these findings provide partial information regarding exploration 2b and how type of education may potentially interact with intervention attitude effects.

### Ethnic-Racial Identity Development and Associated Change in Outgroup and Diversity Attitudes

Main tests of Hypothesis 3, ethnic-racial identity exploration and resolution change and its association with change in outgroup and diversity attitudes, did not reveal any interaction between T1 and T4 when measured using the Ethnic Identity Scale – Brief (EIS-B) (*S*_*L(outgroup)*_ = 0.00, *p* = 0.12; *S*_*L*(acceptance)_ = 0.00, *p* = 0.07; *S*_*L*(respect)_ = 0.00, *p* = 0.46; *S*_*L*(appreciation)_ = 0.00, *p* = < 0.01; for model, see https://osf.io/vb542). These analyses were not run with intervention and control group as predictors of effect, and thus included all adolescents in the sample. Slopes for ethnic-racial identity exploration when measured through the Multigroup Ethnic Identity Measure (MEIM), also did not reveal any longitudinal association in outgroup or diversity attitudes between T1 and T4 (*S*_*L(outgroup)*_ = 0.00, *p* = 0.55; *S*_*L*(acceptance)_ = 0.00, *p* = 0.92; *S*_*L*(respect)_ = 0.00, *p* = 0.86; *S*_*L*(appreciation)_ = 0.00, *p* = 0.67). Tests concerning ethnic-racial identity resolution change and change in outgroup and diversity attitudes was also not able to find any association between T1 and T4 (*S*_*L(outgroup)*_ = 0.00, *p* = 0.47; *S*_*L*(acceptance)_ = 0.00, *p* = 0.16; *S*_*L*(respect)_ = 0.00, *p* = < 0.05; *S*_*L*(appreciation)_ = 0.00, *p* = 0.067). Together, these results fail to support Hypothesis 3, that ethnic-racial identity exploration and resolution growth were associated with growth in outgroup and diversity attitudes.

Follow-up tests of co-variances at individual time points revealed that when ethnic-racial identity exploration was measured using the EIS-B, higher scores on ethnic-racial identity exploration co-varied with higher scores on acceptance of diversity (*S*_*L*_ = 0.05, *p* = <0.05) and respect for diversity (*S*_*L*_ = 0.06, *p* = <0.05) at one time point: T3, regardless of assignment to the intervention or control group. In the same way, higher scores on ethnic-racial identity resolution co-varied with higher scores on acceptance of diversity (*S*_*L*_ = 0.06, *p* = <0.05), respect for diversity (*S*_*L*_ = 0.08, *p* = <0.01), and appreciation of diversity (*S*_*L*_ = 0.06, *p* = <0.05) only at T3. The results thus fail to support Hypothesis 3, but showed significant co-variations between diversity attitudes and ethnic-racial identity exploration and resolution at one time point (T3) when measured using the EIS-B. This co-variation was not reproduced at any of the other time points (T1, T2, or T4), and cannot on its own support the theory of growth in ethnic-racial identity exploration or resolution being associated with growth in outgroup and diversity attitudes.

### Supplementary Analyses

Supplementary, non-pre-registered, analyses were run in order to assess the robustness of the results. Due to baseline differences, one extra, unregistered exploratory latent growth curve model analysis was performed to examine possible interaction effects between migration background and belonging to the intervention/control group. The analysis revealed an interaction effect (*S*_*L(outgroup)*_ = −0.006, *p* = < 0.01) between migration background status and control group status, indicating a slightly larger decrease in outgroup attitudes among adolescents with a non-migration background in the intervention group. Although it reflects only a minor effect in slope, this interaction points toward adolescents with a non-migration background in the intervention group having a slightly larger negative change in outgroup attitudes compared to adolescents with a non-migration background in the control group. An extra, unregistered exploratory latent growth curve model analysis was also used to examine possible interaction effects between type of education and belonging to the intervention/control group but found no differences in terms of either outgroup or diversity attitudes.

Due to high correlations and few items between the three diversity attitude variables (acceptance, respect, and appreciation of diversity), these variables were also aggregated throughout analyses, leading to more stability. The last time point for the control group was handled in different ways to ensure that letting the models predict T4 scores would not produce unreliable results for different groups. Aside from the time-varying factor approach described under preliminary analyses, all analyses were also run T1-T3 as a test of robustness. While second decimals changed for some growth models, this produced no new significant results, and they were always in the same direction as when using the models presented in the results section.

## Discussion

There is little research on whether targeting ethnic-racial identity development can be effectively used to promote positive outgroup and diversity attitudes, something which holds true for adolescents in Sweden; an understudied, culturally diverse setting where demographics have changed rapidly over the last two decades (Statistics Sweden, 2023a). The main aim of this pre-registered study was to examine if an intervention targeting ethnic-racial identity exploration, the Identity Project, also had effects on adolescents’ outgroup and diversity attitudes. The study’s second aim was to examine whether there were differing developmental trajectories for adolescents regarding change in outgroup and diversity attitudes when grouped by migration background or type of education. The third aim of the study was to examine whether positive change in ethnic-racial identity exploration and resolution was associated with positive change in outgroup and diversity attitudes. No positive change in these attitudes was found for either the experiment or the control group and growth models revealed stable or slightly declining slopes across groups. Increases in ethnic-racial identity exploration and resolution co-varied with increases in diversity attitudes at T3, but this effect was not reproduced at the other three time points. Findings from the current study thus indicate that, even when ethnic-racial identity exploration and resolution increased as a function of the Identity Project intervention, positive effects on adolescent outgroup and diversity attitudes did not follow.

### The Identity Project and Attitude Change

While hypothesized to do so, the results do not show that targeting ethnic-racial identity development, via the Identity Project intervention, has a short-to-medium-term impact (26 weeks) on outgroup and diversity attitudes. One possible explanation for why adolescents who received the intervention in Sweden did not show these positive effects is the fact that it is not designed to primarily affect adolescents’ outgroup and diversity attitudes, even if cascading effects on outgroup attitudes (ethnic-racial identity exploration initiating processes of outgroup attitude change) have been found in the US (Umaña-Taylor et al., 2018), and there was cross-sectional support for the relationship in a German Identity Project study (Juang et al., 2020). One reason for why these results were not replicated in the Swedish sample could be that the sociocultural adaptation of the intervention (Juang et al., 2023) made it different in ways that did not result in positive outgroup and diversity attitude change. However, the Swedish adaptation of the intervention did indeed mostly work as intended, as main effects for ethnic-racial identity exploration and partial effects on resolution were found in the sample (Abdullahi et al., 2024). When it comes to differences and similarities between studies in different countries, post-intervention scores on outgroup attitudes do not differ much between the US (OGO_T4_ = 3.29), German (OGO_T3_ = 3.31), and Swedish (OGO_T4_ = 3.19) samples. Interestingly, the Swedish sample reported higher mean scores on attitudes at baseline (OGO_T1_ = 3.33) than adolescents in other countries did post-intervention, painting an interesting picture of the impact of the Swedish sociocultural context. Unfortunately, no such international comparisons can be made concerning diversity attitudes, as the scale has not previously been used with the Identity Project. Furthermore, high scores on attitudes (e.g., OGO_T1_ = 3.33, range 1–4; Acceptance of Diversity_T1_ = 4.37, range 1–5) do not necessarily translate to actual positive outgroup behaviors, or a positive and welcoming society for that matter, so a careful approach is needed when interpreting and comparing such results.

Another reason why the Identity Project did not have a positive impact on outgroup and diversity attitudes could have to do with how engaging in reflection on or discussion of sensitive subjects such as discrimination, stereotyping, and racism may have increased awareness among adolescents regarding unfair treatment and the presence of systems and institutions that can perpetuate inequities. Findings from previous research do indicate that identification can be linked to perceived outgroup threat (Pettigrew et al., 2007), but also that this link can be attenuated with increased identity exploration, painting a complex picture where both content and context factors matter (Spiegler et al., 2022). The Identity Project may, then, have prompted increased critical consciousness and perceived outgroup threat, possibly limiting a positive impact on adolescents’ attitudes toward outgroups and cultural diversity. Such changes in perceived outgroup threat or critical consciousness are of interest for interventions using identity exploration to improve attitudes, as this suggests that learning about one’s group membership might, at least temporarily, increase one’s susceptibility to forms of perceived outgroup threat, possibly accounting for some of the slight declines in attitude slopes. Another interesting way to look at this is through an intergroup relations perspective, in which adolescents may find many different ways to relate to outgroups and cultural diversity when presented with it, either intuitively or deliberately. They may for example consider reasons for tolerating what they disapprove of, often referred to as forbearance tolerance (Verkuyten et al., 2022), or may simply seek to reject these differences, leading to increased intolerance. Taking the Swedish sociocultural context into account, diversity is also a relatively new phenomenon in comparison to the US, meaning that many groups have a short history of living together in the same environment, an aspect which could have influenced how the intervention impacted (or rather did not impact) outgroup and diversity attitudes for the intervention group as a whole.

The time frame of the intervention is yet another vital aspect to address. Twenty-six weeks between baseline and last measurement is possibly too short a period to see more substantial changes in values or attitudes. The last follow-up measurement in the original US intervention was 67 weeks after baseline, meaning that both sleeper and maturation effects could be relevant and may explain the fact that attitudes did not change among the adolescents in the Swedish version. Consequently, it will be necessary to conduct similar statistical analyses with longer-term follow-ups to ascertain potential delayed positive outcomes. Another important aspect to discuss is the age of the adolescents who participated in the intervention. In the Swedish adaptation of the intervention the adolescents were one to three years older than those in other countries’ interventions (Ceccon et al., 2023; Juang et al., 2020; Umaña-Taylor et al., 2018). This decision to include older adolescents was primarily related to COVID-19 regulations in Sweden and the fact that high schools still allowed researchers on their premises. It may be the case that positive change in outgroup and diversity attitudes is not as prominent later in adolescence, or that the students in the current intervention had had more time to explore their attitudes when the intervention occurred. One last possible explanation for the difference in findings between this study and those from the US and Germany is that the adolescents in the Swedish study might have been primed by external events either at, before, or after baseline, leading to negative attitudinal shifts within both the intervention and control groups. While this is speculatory, such priming could have occurred through increasingly polarized political discussions in the context, educational changes-, or decreased intergroup contact due to COVID-19 regulations. In summary, while there are possible reasons as to why, this study does not provide support for a link between ethnic-racial identity development and positive outgroup and diversity attitudes when facilitated via the Identity Project intervention.

### Different Trajectories of Change in Attitudes

This study also examined whether adolescents’ migration backgrounds could impact the trajectories of change, but found no difference in intervention effect on attitudes among those with migration backgrounds compared to those without migration backgrounds (Hypothesis 2a). There was, however, an interaction effect whereby participants in the non-migration group decreased more in attitudes when they were also part of the intervention group. Looking at individual trajectories only, adolescents with a migration background have a slightly more stable attitude pattern. Similarly, individual trajectories in the data revealed that adolescents from the non-migration background group initially scored higher on outgroup and diversity attitudes but declined more over time. However, as this is only based on individual slopes for migration and non-migration background groups (rather than a significant difference between the two groups) –these are not reliable differences in attitude trajectories. One possible interpretation regarding how the migration background group seems more stable in terms of attitudes while the non-migration background group may move more is that the migration background group may have already explored their own ethnic-racial identity and attitudes to a greater extent (Syed & Azmitia, 2009). This is also in line with previous research showing that majority, non-immigrant adolescents are less likely to have explored their ethnic-racial identity than are minoritized, migrant adolescents, likely due to their position within the societal norms (Galliher et al., 2017). This result also makes sense in relation to social identity theory, which emphasizes how different parts of the identity become salient and relevant in different contexts and at different times (Tajfel & Turner, 1979), a phenomenon of relevance to adolescents’ attitude development.

Regarding adolescents’ type of education – theoretical or practical education programs – the results show that adolescents in theoretical programs scored higher on outgroup attitudes at baseline and had larger, individual declines in only one out of four attitude measures, when compared to adolescents in practical education programs. While these results could imply that hypothesized cascading effects of the Identity Project, such as attitudes change, may be impacted by factors such as the type of education program that adolescents are enrolled in, the differences in trajectories are minor. This is similar to findings in previous studies on outgroup attitudes in Sweden, which showed different developmental trajectories in positive attitude for adolescents in different types of education (Lundberg & Abdelzadeh, 2017). In summary, while migration background status did not predict any difference in attitude trajectories, type of education could be important from the perspective of intervention work, and might be beneficial to consider when tailoring interventions to better target adolescents in different types of educational settings.

### Ethnic-racial Identity Development and Attitude Change

Regarding the relation between ethnic-racial identity and attitudes, it was hypothesized that a change in ethnic-racial identity exploration and resolution would be the motor driving change in outgroup and diversity attitudes (Hypothesis 3). This relation was not supported in the data. However, ethnic-racial identity exploration and ethnic-racial identity resolution co-varied with diversity attitudes at T3, which may indicate some potential for association between the two. While it is interesting that changes in ethnic-racial identity moved in conjunction with changes in attitudes at one-time point, it is not certain that one is associated with the other, as there was not enough covariance in the variables over all four time points. Why this effect occurs at T3 could be due to chance, but T3 was also a time point at which ethnic-racial identity exploration had increased within the sample, and possible cascading effects such as attitudes might have set in.

Another possible explanation for the lack of association over time points is that several different aspects of ethnic-racial identity exploration and ethnic-racial identity resolution may relate to attitudes in different ways, and that some of these aspects of ethnic-racial identity did not change during the intervention. Different parts of ethnic-racial identity development – such as how central, well regarded, or affirmed one’s ethnic-racial identity is – could be more or less impactful for change in outgroup and diversity attitudes. The intervention is seemingly specifically targeted at ethnic-racial identity exploration participation; and analyses with the same dataset as this study found little development in terms of other related constructs such as ethnic-racial identity exploration search, ethnic-racial identity centrality, or ethnic-racial identity private regard (Abdullahi et al., 2024). Although this is merely speculation, such aspects of ethnic-racial identity development might help explain the lack of positive findings regarding outgroup and diversity attitudes, and is something that future studies could further investigate. Future research may also benefit from further exploring the interaction between ethnic-racial identity and attitudes, using both quantitative and qualitative methods, and doing so in multiple contexts and age groups. In summary, while previous research points to an interaction effect between ethnic-racial identity development and outgroup and diversity attitudes, this study provides no such definitive support.

### Limitations

There are several limitations to this study, an important one being the disruptive nature of the COVID-19 pandemic. The intervention was facilitated in schools at a point in the pandemic when infection rates were very high. Most high schools in Sweden remained open during the pandemic, in contrast to schools in many other countries that resorted to homeschooling for this age group. Despite the Swedish schools being open, the pandemic still affected adolescents’ psychosocial lives in many ways (Germani et al., 2020). The facilitation of the intervention was also impacted, the most notable aspect being facilitators and adolescents falling ill and abiding by quarantine rules (e.g., staying at home and being unable to attend classes if oneself or one’s family had symptoms). This in turn could have impacted the efficacy of the intervention. It is also possible that adolescents’ intergroup contact, previously linked to increased outgroup attitudes (Tropp & Pettigrew, 2005), was diminished during the pandemic, and that this could have impacted the results. One more limitation is how the division of migration and non-migration background amongst the adolescents in the sample is unprecise. While many share the experience of belonging to an ethnic minority within Swedish society, the division may not properly reflect change for certain individuals or groups within this highly diverse cluster.

Another possible limitation is the psychometric properties of measurements in different contexts. With adolescents in this study scoring high on both outgroup and diversity attitudes at baseline, two issues that might have affected the results need to be considered: ceiling effects and statistical regressions toward the mean. Ceiling effects can occur when a significant proportion of data points in a dataset reach the higher end of possible scores, limiting the ability to differentiate between adolescents at the upper end of the scale and potentially obscuring meaningful variations or differences in scores, in this case, attitudes (Cramer & Howitt, 2004). Also, in any longitudinal sample in which scores on a variable are far from the scale mean at one-time point, scores at the following time point (in this case, the subsequent measurement) are statistically more likely to be closer to the scale mean (Everitt & Skrondal, 2010). These two limitations, alone or in combination, might account for some of the small, negative slopes visible across all attitude measurements. However, there is some evidence against this type of phenomenon being the sole driving factor of negative change, as the control group started higher than the intervention group on outgroup attitudes but showed less decrease over time. Another issue to consider is the reliability scores of the OGO measure being slightly below adequate. One possible way to address this in future studies, if backed by analyses such as confirmatory factor analyses, is to separate the OGO measure into a two-factor structure: intergroup approach and intergroup avoidance (Satterthwaite-Freiman et al., 2022; Wantchekon et al., 2023). Lastly, it is possible that the adolescents’ initial high scores on attitudes in the sample stem from social desirability biases that are more present in the Swedish context than in other countries, in turn leading to inflated scores and attitudes having little room to increase.

## Conclusion

While there are many well-researched, positive aspects of ethnic-racial identity development for adolescents, little is known about its function as a pathway to outgroup and diversity attitudes. This study investigated whether targeting adolescents’ ethnic-racial identity exploration via an intervention, the Identity Project, also resulted in positive outgroup and diversity attitudes in the context of Sweden. The findings indicate that such positive effects on attitudes are not a given, even when ethnic-racial identity exploration and resolution increased for those partaking in the intervention. No positive longitudinal attitude change was found for the intervention or control group. Similarly, no positive longitudinal change was found when adolescents were grouped by migration background, or by type of education. Positive change in ethnic-racial identity development was not associated with positive change in outgroup and diversity attitudes over time but showed a small indicator of co-variation between the two at one-time point, Time 3. The results thus complicate the notion of outgroup and cultural diversity attitude change as a cascading effect of targeting ethnic-racial identity development. This raises important questions regarding effective strategies for promoting positive outgroup and diversity attitudes amongst adolescents in diverse cultural settings around the globe.
